# Convolutional Neural Network Applied to SARS-CoV-2 Sequence Classification

**DOI:** 10.3390/s22155730

**Published:** 2022-07-31

**Authors:** Gabriel B. M. Câmara, Maria G. F. Coutinho, Lucileide M. D. da Silva, Walter V. do N. Gadelha, Matheus F. Torquato, Raquel de M. Barbosa, Marcelo A. C. Fernandes

**Affiliations:** 1 Bioinformatics Multidisciplinary Environment (BioME), Federal University of Rio Grande do Norte, Natal 59078-970, RN, Brazil; gmottaz@gmail.com; 2 Laboratory of Machine Learning and Intelligent Instrumentation, Federal University of Rio Grande do Norte, Natal 59078-970, RN, Brazil; gracielly@dca.ufrn.br (M.G.F.C.); lucileide.dantas@escolar.ifrn.edu.br (L.M.D.d.S.); wgadelha@gmail.com (W.V.d.N.G.); matheusft@dca.ufrn.br (M.F.T.); 3 Federal Institute of Education, Science and Technology of Rio Grande do Norte, Paraiso, Santa Cruz 59200-000, RN, Brazil; 4 Department of Pharmacy and Pharmaceutical Technology, University of Granada, 18071 Granada, Spain; 5 Department of Computer Engineering and Automation, Federal University of Rio Grande do Norte, Natal 59078-970, RN, Brazil

**Keywords:** SARS-CoV-2, COVID-19, deep learning, CNN

## Abstract

COVID-19, the illness caused by the severe acute respiratory syndrome coronavirus 2 (SARS-CoV-2) virus belonging to the *Coronaviridade* family, a single-strand positive-sense RNA genome, has been spreading around the world and has been declared a pandemic by the World Health Organization. On 17 January 2022, there were more than 329 million cases, with more than 5.5 million deaths. Although COVID-19 has a low mortality rate, its high capacities for contamination, spread, and mutation worry the authorities, especially after the emergence of the Omicron variant, which has a high transmission capacity and can more easily contaminate even vaccinated people. Such outbreaks require elucidation of the taxonomic classification and origin of the virus (SARS-CoV-2) from the genomic sequence for strategic planning, containment, and treatment of the disease. Thus, this work proposes a high-accuracy technique to classify viruses and other organisms from a genome sequence using a deep learning convolutional neural network (CNN). Unlike the other literature, the proposed approach does not limit the length of the genome sequence. The results show that the novel proposal accurately distinguishes SARS-CoV-2 from the sequences of other viruses. The results were obtained from 1557 instances of SARS-CoV-2 from the National Center for Biotechnology Information (NCBI) and 14,684 different viruses from the Virus-Host DB. As a CNN has several changeable parameters, the tests were performed with forty-eight different architectures; the best of these had an accuracy of 91.94 ± 2.62% in classifying viruses into their realms correctly, in addition to 100% accuracy in classifying SARS-CoV-2 into its respective realm, *Riboviria*. For the subsequent classifications (family, genera, and subgenus), this accuracy increased, which shows that the proposed architecture may be viable in the classification of the virus that causes COVID-19.

## 1. Introduction

The *Coronaviridae* family has been characterized as a (+) ssRNA (positive-sense single-stranded RNA virus) and has been identified in avian and mammalian hosts (including humans). This virus family has a genome length that ranges between 26 and 32 k base pairs (kbp). In humans, the *Coronaviridae* family includes viruses such as SARS-CoV, MERS-CoV, HCoV-OC43, HCoV-229E, HCoV-NL63, and HCoV-HKU1 [1,2].

The new virus from this family, named SARS-CoV-2, is responsible for the COVID-19 illness and was discovered in Wuhan, China [3,4]. This virus has a great affinity for the angiotensin-converting enzyme 2 receptor [5], using it as the main site of penetration into a host cell due to its affinity for the spike (S) protein; it is translated by at least one of the six ORFs of the viral genome [6,7]. Genomic similarity studies show similarities with viruses from bats, which is the probable origin of it, although other animals, such as the pangolin, are also candidates [8,9]. Ref. [10]: such outbreaks demand elucidation of the taxonomic classification and origin of the virus’s genomic sequence for strategic planning, containment, and treatment.

To address the challenges associated with identifying and classifying all species, several techniques have been proposed for the analysis and comparison of genomic sequences. These methods are usually categorized as with or without alignment.

Classification techniques applied to viral sequences have been based on alignment methods or algorithms, such as the Needleman–Wunsch [11], Smith–Waterman [12], FASTA [13], BLAST [14,15], and others. These methods use different techniques, such as dynamic programming, pairwise comparison, and heuristic methods associated with similarity metrics between the nucleotide sequences. However, these methods have some limitations: (1) They require some prior knowledge of the reference sequence; (2) they admit that there is contiguity between homologous regions; (3) they are computationally expensive for long sequences (for example, for only two sequences of length N, we have (2N)!/${\left(N!\right)}^{2}$) possible gapped sequences and a time complexity of the length of the inputs and/or their products; and (4) they are not very efficient for specimens that have a high rate of genomic mutation, such as viruses [16,17,18,19,20].

In order to mitigate these limitations, new alignment-free methods (AFMs) have been explored. Normally, the AFMs are composed of two cascaded stages called mapping and processing, respectively. In the mapping stage, the sequence is mapped to a new representation space, called feature space [16,17]. There are many different mapping strategies to produce the feature space, such as word composition, entropy, signal space, and others [21,22,23,24,25,26,27,28]. In some cases, multiple mapping strategies are applied sequentially. The research presented in [16,17] details several of these strategies.

In the processing stage, the AFMs can use clustering or prediction techniques, depending on the application [16,17]. For the first option, the AFMs use a similarity metric to cluster the information. Applications such as host prediction, phylogenetic tree, genotype–phenotype association, and others can use this approach and many different similarity metric schemes [16,17]. On the other hand, for applications such as gene interaction prediction, protein classification, drug repositioning, genome classification, and others, prediction techniques can be applied. The work presented in [18,19,20] compares multiple AFMs’ approaches.

Several works in the literature explored the use of machine learning (ML) algorithms and techniques in the processing stage [10,16,17,25,27,28,29,30,31,32,33,34,35,36]. It can be noted that, among these applications, deep learning (DL) is the most frequently used approach [28,31,32,33,34,35,36]. Deep learning is part of a broader family of machine learning methods based on artificial neural networks (NN) that are capable of identifying highly complex patterns in large datasets [34,35,36].

Thus, the present work proposes a new alignment-free approach based on deep learning to classify genomic virus sequences. In this proposal, AFM-2D*k*MCNN uses the *k*-mers 2D image technique in the mapping stage and convolutional neural networks (CNN) in the processing stage. The genome sequence classification has proved to be an important approach for the identification of genomic variability, taxonomic aspects, and disease mechanisms. Comparative genomic analyses can benefit significantly from AFM tools based on DL as they are generic, fast, and accurate in classifying newly sequenced strains from several virus families [25,30,33].

Previous works [37,38,39,40,41] have proposed genome classification strategies that use alignment-free methods with a CNN in the processing stage. The research presented in [37] shows a deep learning neural network for DNA sequence classification. The proposals presented in [37] used a spectral sequence representation (*k*-mers representation) in the mapping stage and LeNet-5 CNN in the processing stage. It was tested with 16S rRNA sequences, and the performances (in terms of accuracy and the F1 score) were compared to other machine learning techniques, such as regression neural network, naive Bayes, random forest, and support vector machine classifiers. In this work, the input sequence size was limited to 1200 bp. A DL applied to DNA classification was also proposed in [38]. In this work, the authors used a one-hot vector representation of DNA sequence in the mapping stage and a CNN for processing. The proposal was limited to sequences with a maximum length of 500 bp.

In [39], a deep learning-based method, called the ViraMiner, was developed to identify viruses in various human specimens. The ViraMiner uses the one-hot vector representation and a CNN in the mapping and processing stages, respectively. The processing stage contains two branches of a CNN designed to detect both patterns and pattern frequencies on raw metagenomic contigs. The training dataset included sequences obtained from 19 metagenomic experiments, which were analyzed and labeled by the BLAST algorithm. The ViraMiner works with sequences of 300 bp.

Similarly, the work presented in [40] proposed a method called DeepVirFinder. DeepVirFinder used the same approach as the ViraMiner, presented in [39]; however, it can work with 300, 500, 1000, and 3000 base pairs, and it outperformed the ViraMiner in terms of accuracy.

A deep learning genome classification strategy targeting SARS-CoV-2 was proposed in [41]. Following a different approach from the work presented in [37,38,39,40], this research is capable of working with 31,029 bp (maximum length of available cDNA sequences for a coronavirus) [1]. The work presented in [41] used the direct number representation [24] and a CNN in the mapping and processing stages, respectively.

The work presented in [42] also used RNA as the input in the form of a one-hot encoding; however, a tree-shaped model in which each step classifies the virus into a taxon and advances it on to the subsequent taxon classification was used, and, in the end, a genetic sequence may be even excluded as viral RNA.

The work proposed in this paper consists of a representation of the genetic code of SARS-CoV-2 in image form, using the frequency of its *k*-mers, which are substrings of size k of the genetic code. These images will be submitted to a CNN for viral classification, mainly of SARS-CoV-2. The proposed *k*-mers’ images, associated with the CNN, make up a faster alignment-free method of comparing sequences than the methods described in this section. This approach is less susceptible to rapid evolutionary changes in the viral genome, thereby generating more accurate and reliable results, especially in RNA viruses. On the other hand, the k-mers’ methods are susceptible to the choice of *k* size, which requires testing new imaging methods as the CNN’s input. Thus, this work brings the following specific contributions:

- Develop and validate a new genomic viral classification based on the k-mers’ image representation and a CNN.
- Develop an alignment-free method to classify SARS-CoV-2 sequences as viruses from different realms, families, genera, and subgenera.
- Develop a deep learning algorithm that can efficiently classify the complete cDNA sequences of the virus.

## 2. Materials and Methods

### 2.1. Data

The conducted experiments used a database of 1553 SARS-CoV-2 RNA samples (Dataset1) downloaded from the National Center for Biotechnology Information (NCBI) and 14,684 samples of other viruses downloaded from the Virus-Host DB (Dataset2). For each experiment performed, sub-datasets were created, called Dataset(3–6). Complete descriptions of Dataset1 and Dataset2 are in Table 1. Some of the viruses do not have a taxon classification, e.g., the virus with code in the Virus-Host DB of code NC_043374 has no classification within any realms but does have classifications in the other taxa. When this occurs, it is called unclassified and will not be considered in the analysis for that taxon. For the subgenus taxon, almost all samples are unclassified.

### 2.2. Image Representation

**Figure 1:**
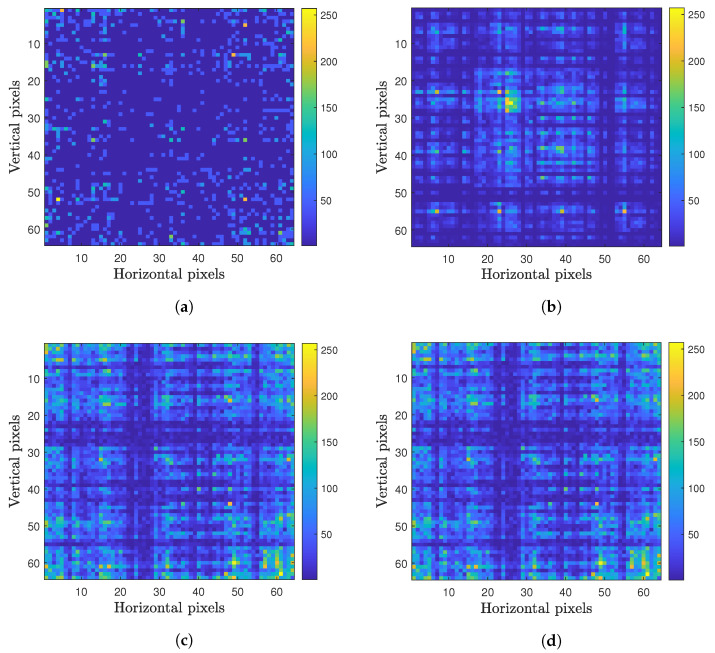
Examples of viral genomes in the form of images created by the process described in Section 2.2: (**a**) Paramecium bursaria Chlorella virus (NC_043234); (**b**) Gordonia phage Obliviate (NC_031237); (**c**) SARS-CoV-2 USA 2020 (MT251977); and (**d**) SARS-CoV-2 Wuhan-Hu-1 (MN908947).

A representation of the viral genome was used in the form of its *k*-mer, which is a substring of the genetic code in question of a size *k* with a maximum size of L−k+1, where *L* is the size of the genetic sequence (about thirty thousand base pairs (bp) for SARS-CoV-2), and $n^{k}$ is the possible *k*-mers, where *k* is the number of possible monomers; in this case, they are k=4, one for each nitrogen base of DNA. In this work, of all the *k*-mers chosen, the one that presented the best result for a CNN’s accuracy was 6. The *k*-mer equal to k=6 was chosen because it generates highly accurate results with a short network training time. When the *k*-mers are equal to or greater than 7 have little impact on increasing the accuracy but greatly increase the training time. This brings us to a maximum number of 64 *k*-mers, which will be used throughout this work. To form the sets of images, each one represents a virus that will be used as an input to the CNN. The frequency with which each *k*-mer appears in the genetic code of a virus becomes a pixel of a 64×64 pixel image (64 horizontal pixels and 64 vertical pixels). Figure 1 shows four examples of image representations of the viral genome. Each one of these images represents a specific ”fingerprint” of the genetic code of a particular virus. As some *k*-mers appear with a much higher frequency than others, which could generate large discrepancies in the images that would make classification by a CNN difficult, a normalization of the values of each pixel was performed by the highest *k*-mers’ frequency value. The result of this process can be seen in Figure 1. The mathematical process behind this methodology was previously described by [43], and a dataset with several examples can be found in [44].

### 2.3. Processing

Deep learning has proved its efficiency over fellow state-of-the-art image classification techniques. It uses layered algorithms to perform the recognition of patterns. Its recognition is refined through each layer, until the last one, upon which the image is finally classified.

The deep learning approach presented in this paper is based on the convolutional neural network (CNN). The CNN is a supervised method of learning with several layers, inspired by visual cortex biologic cells. Cortical neurons respond to stimulation only per a region of their field of view, and then imbricate them, reaching the whole visual field [45]. Convolutional layers are the main structure of a CNN network. The layers are composed of a set of learnable filters (or kernels). Each filter is activated to detect a specific sort of feature in a small region of the input (or feature map). The filters’ activation maps, bundled in depth, form the output of the convolutional layer. The outcome is crossed to the following layer, similar to what a virtual cortex does when it receives a stimulus. In addition to the convolutional layers, the network is also composed of a rectified linear unit (ReLU) as the activation function, max pooling layers for size reduction, and fully connected layers for classification.

**Figure 2:**
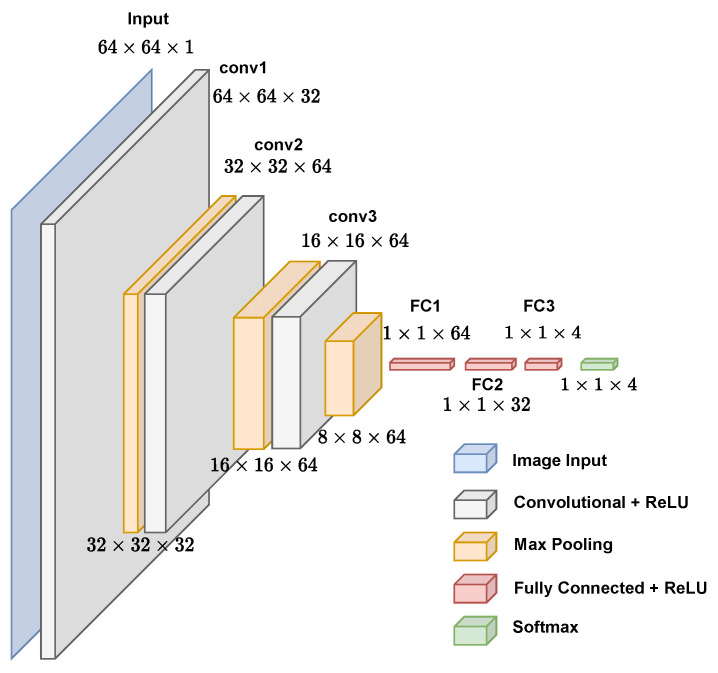
Graphical representation of proposed convolutional neural network for virus genome classification.

The inputs of the CNN used in this paper are sets of images of 64×64 pixels, in which each one represents the frequency of a *k*-mers within the genomic sequence of a virus (see Section 2.2). The arrangement of the following layers is shown in Figure 2. The first convolutional layer has 32 filters with a 7×7 filter size. The second convolutional layer has 64 filters with a 5×5 filter size, and the third convolutional layer has 64 filters with a 3×3 filter size. The choices of filters and the number of layers were made based on the training of several different architectures; the ones that are shown here obtained greater accuracies in their training and testing with lower complexities; therefore, architectures with filters greater than 7×7 and 4 or more convolutional layers were not chosen because they do not interfere with the model’s accuracy. All of these have a stride of 2 and padding to keep the input image’s dimensions unmodified, followed by a rectified linear unit (ReLU) layer and max pooling layer with a 2×2 kernel. In the end, there are 2 fully connected layers: one with 64 neurons and the other with 32 neurons. Each one has a 40% dropout (smaller values were tested but decreased the accuracy of the model). For classification, there is a fully connected layer with an equal number of neurons to the number of classes used in each level of the taxonomic classification (realm, family, genus, and subgenus). For example, for the classification of viruses in realms, there were 4 neurons in this layer, which is equivalent to the 4 possible realms: *Monodnaviria*, *Riboviria*, *Duplodnaviria*, and *Varidnaviria*.

The realms have the greatest differences from each other because a realm is the broadest taxon within the viral classification; thus, it was chosen for the validation and training of the network, as it is expected that the accuracy resulting from the experiment with the realms does not decrease as much for the realms as for the others, which have more similarities to one another.

Due to the large number of possible CNN architectures, forty-eight variations of network parameters were first tested, in order to lead us to choose the one that presents the best accuracy in classifying viruses into their correct taxa. If the accuracy values of a given architecture are close to those of another, then the fastest and least computationally expensive one will be preferred. The following parameters were evaluated for the CNN: three possibilities for input normalization; zero–one input images, in which they are normalized to the interval {0,1}; symmetry, for which the interval is {−1,1}; mean, in which the average of the values is subtracted from each pixel; mini-batch size, with a size of 64 or 128 samples; the configuration of the 2D filters of each layer with possible configurations of 7×7−5×5−3×3 or 5×5−5×5−3×3; the stride of each layer possibly being one or two; and the cross-validation used possibly being able to divide the dataset used in 90% of the samples for training and 10% for validation (10-fold) or 80% for training and 20% for validation (5-fold). The optimization algorithm used was the Adam with an initial learning rate of 0.001 and a validation frequency of 8, and a maximum number of epochs of 60 was chosen if, after it, there is no more variation in the accuracy of the model. The CNN was trained as many times as the number of the folds used, and the mean and standard deviation of the accuracy were calculated. Forty-eight different architectures were tested, and the results of the mean accuracies of the validation and standard deviation for the 5 best results are shown in Table 2.

## 3. Results and Discussion

### 3.1. CNN Architecture, Confusion Matrices, and Histograms

**Figure 3:**
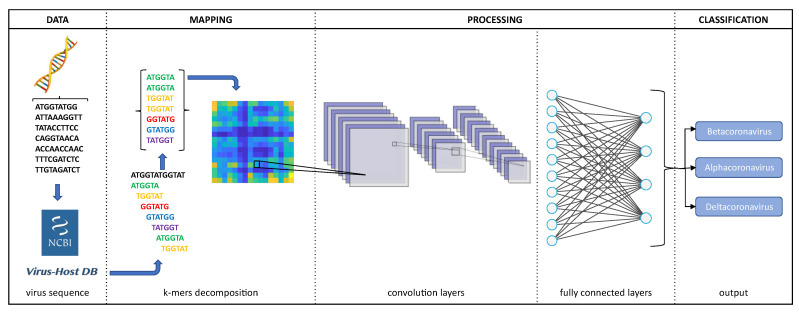
Overview of the proposed approach.

As seen in Table 2, the two architectures with the best validation accuracy values had the same filter sizes in the 7×7−5×5−3×3 option (processing area of Figure 3); normalizations of the zero–one type input; and mini-batch sizes of 128, which represent a speed gain compared to 64 since it takes less time to present all samples to the network. As for cross-validation, the best results were from partitioning the group into 10-fold, which presents a greater execution time than that of 5-fold, but does not interfere as much as stride changes, and, therefore, the value of 10-fold was used. Finally, the stride had the best result with the value of one, and there was little difference in the accuracy result for the value of two; this little difference is not seen in the execution times of each test, where for stride 1, we have 15.47±1.56 minutes (n=10) and, for stride 2, we have 1.45±0.03 (n=10) (mean ± standard deviation) minutes. To compare the two architectures, a Student’s T-test was performed, which showed that there is no statistical difference between the two accuracy means (p-value=0.9457), so we proceeded with the stride value of two. The final architecture chosen and used henceforward for the network was: an input layer with a dimension of 64×64 pixels and zero–one normalization; three convolutional layers with, respectively, 32, 64, and 64 filters; and a filter size of 7×7, 5×5, and 5×5, respectively, with an activation function of the ReLU type (Rectified Linear Unit), a dropout of two, a stride of two, and the same padding. After the convolutional layers, we have two max pooling layers with 64 and 32 neurons, respectively, and a 40% forgetfulness rate. Finally, we have a fully connected layer and a softmax layer for classification. A 128-sample mini-batch was used, with a *k*-fold of k=10, a learning rate of 0.001, a validation frequency of 8, and the Adam as the optimization algorithm. All sets were trained for 40 epochs.

**Figure 4:**
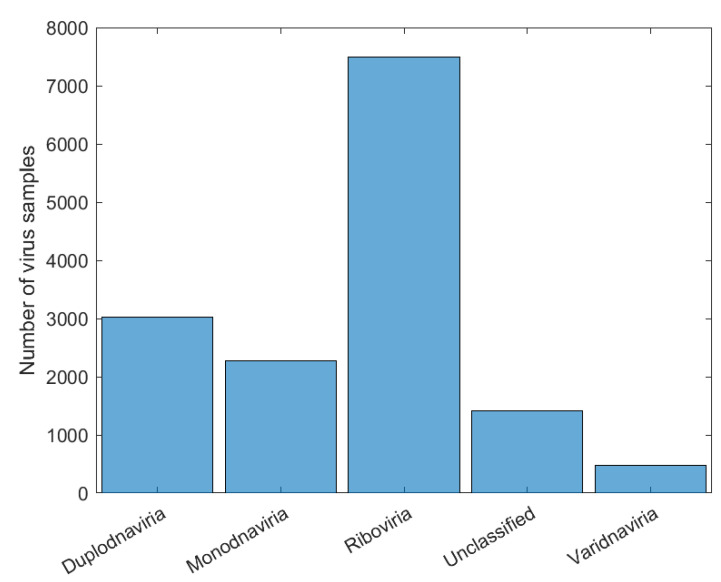
Number of samples in each realm and with unclassified label before treatment.

After choosing the CNN’s architecture, four experiments were carried out (Exp1, Exp2, Exp3, and Exp4), in which, respectively, the viruses were classified based on the realms, families, genera, and subgenera to which they belong. In Exp1, all samples from Dataset2 were used for CNN training and virus classification in four realms: *Duplodnaviria*, *Monodnaviria*, *Riboviria*, and *Varidnvaria*. In Figure 4, we have the histogram of the distribution of the number of samples from each realm in Dataset2, which shows a large discrepancy between the number of samples from the analyzed kingdoms: *Riboviria* (SARS-CoV-2 Realm) has the highest number, 7489, and *Varidnvaria* has the smallest, with 476 samples.

To prevent this difference from leading to overfitting of the network (in which it only recognizes the instances with more samples), 400 viruses were randomly selected from each realm to compose the new training dataset (Dataset3) for Exp1, thus adding 1600 samples in total.

**Figure 5:**
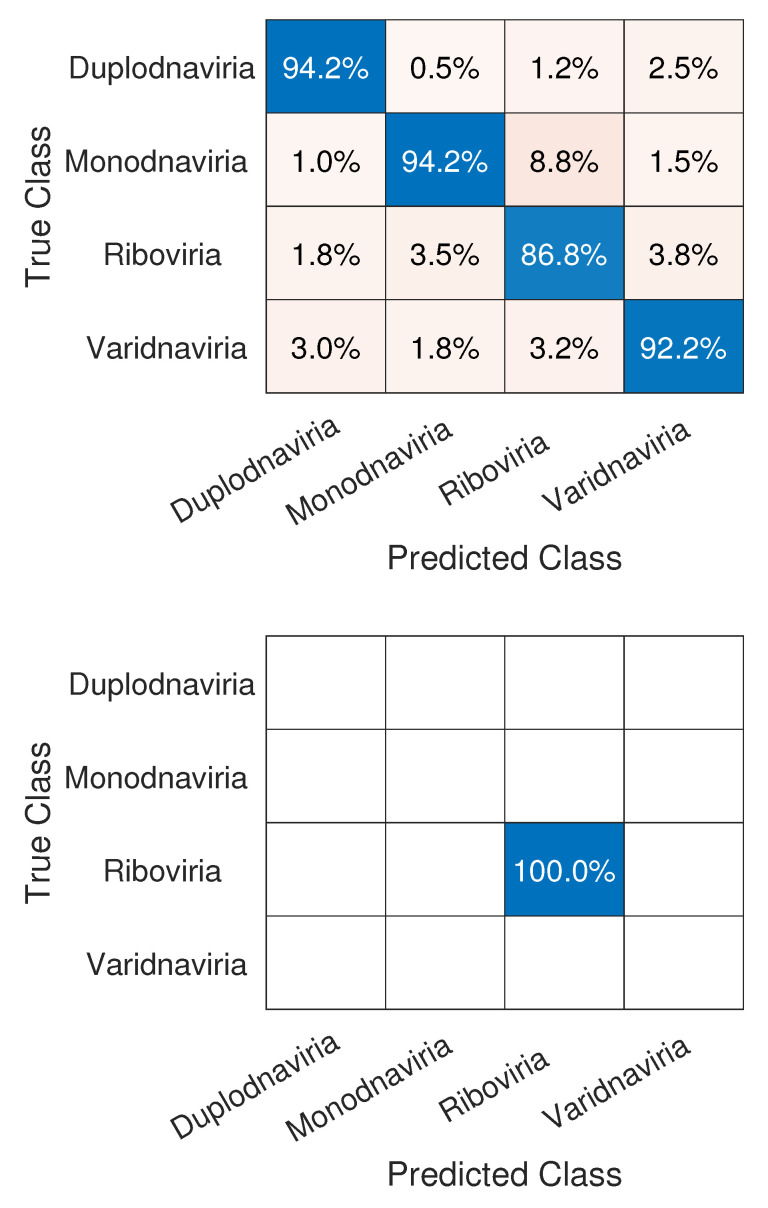
Confusion matrices for Experiment1, which show results for the classification of the viruses in their respective realms (**top**) and SARS-CoV-2 in the *Riboviria* realm (**bottom**).

We can see in Figure 5 that the realm that obtained the lowest accuracy was *Riboviria* (86.8%), a realm that includes RNA and some DNA viruses: double-stranded RNA (dsRNA), positive single-stranded RNA ((+) ssRNA), negative single-stranded RNA ((−)ssRNA), single-stranded RNA with reverse transcriptase (ss RNA-RT), and double-stranded DNA with reverse transcriptase (dsDNA-RT) [46], which are, respectively, groups III, IV, V, VI, and VII of the Baltimore Classification System. Each of the three other realms consists only of viruses with a DNA genome, which may have led to the difference in classification. Regarding the classification of SARS-CoV-2, the network achieved a perfect result with 100% accuracy, therefore correctly classifying it in its true realm, the *Riboviria*.

For the classification of families within those of the *Riboviria* realm (Exp2), it was necessary to choose those with more representatives in Dataset2, as the total number of families is 95. Taking into account the number of samples from each family, we have a median of 18 and approximately 20% of families have less than four samples (which is the value of the first quartile), which shows that a lot of the families have only a few samples and that families such as *Reoviridae* have more than 1000 samples. Because of this, to avoid overfitting, only families with more than 100 samples were selected for training and validation, reaching a total of 16: *Betaflexiviridae*, *Bromoviridae*, *Caliciviridae*, *Coronaviridae*, *Flaviviridae*, *Hantaviridae*, *Orthomyxoviridae*, *Partiviridae*, *Peribunyaviridae*, *Phenuviridae*, *Picoraviridae*, *Potyviridae*, *Reoviridae*, *Retroviridae*, *Rhabdoviridae*, and *Secoviridae*.

**Figure 6:**
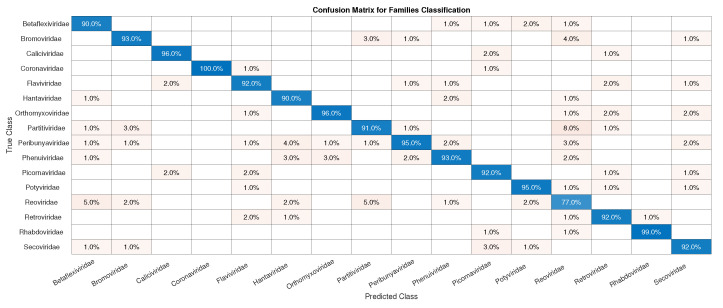
Confusion matrix for validation of the CNN using Dataset4.

**Figure 7:**
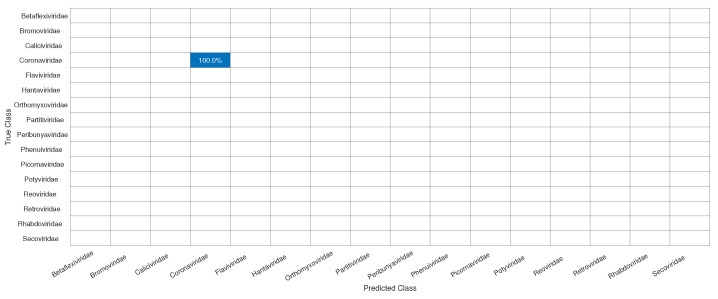
Confusion matrix for the test of the CNN for the classification of the SARS-CoV-2 in its correct family (*Coronaviridae*).

As in Exp1, to avoid overfitting, since we have families such as *Reoviridae*, with more than 1000 samples, and *Picornaviridae* and *Caliviridae*, with more than 400, we randomly chose 100 samples of each to form Dataset4, which was used for training and validation in Exp2, totaling 1600 samples. In Figure 6, we have the confusion matrices for validation of the network using Dataset4, and, in Figure 7, the test was completed with Dataset1.

**Figure 8:**
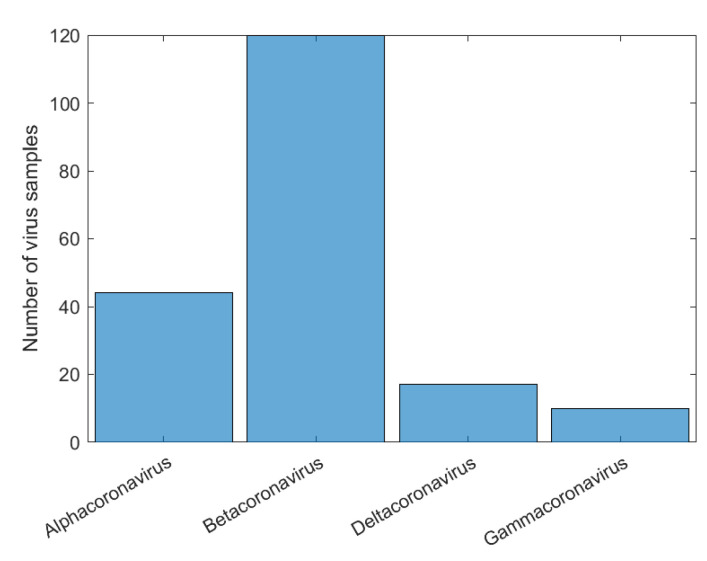
Histogram of sample distribution for the Exp3 before normalization.

**Figure 9:**
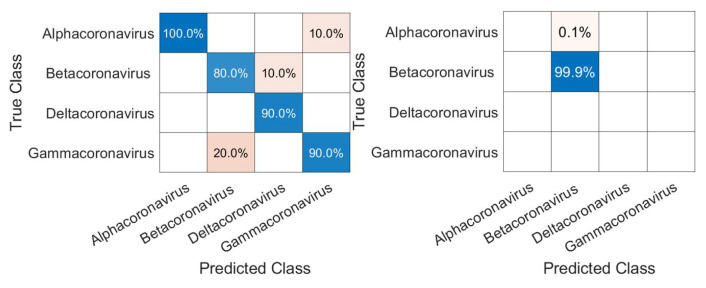
Confusion matrices for validation of the CNN using the genus inside (*Coronaviridae*) the family (**left**) and, for classification of SARS-CoV-2, in its correct genus (*Betacoronavirus*).

Initially, we can see that the CNN correctly classified 100% of SARS-CoV-2 in its family, *Coronaviridae*. For the confusion matrix, it is observed that the *Reoviridae* family presented the lowest accuracy (82%). It is the dsRNA (double-stranded) family with the largest number of genera (12) inside it and reaches a wide range of mammals, plants, birds, reptiles, crustaceans, fish, protists, insects, plants, and fungi. Its genetic code has very different sizes, ranging from 1200 bp to almost 4000 bp, with a constant rearrangement. In addition to the untranslated regions of the genome, these present a great variation, between 12 nt and 83 nt. Further, since the parts conserved between the species are close to the 5’ and 3’ ends, they are not being conserved in other parts of the genome. All of this information shows that the big differences within the family may explain why the CNN had a harder time classifying it [46]. The classification within the *Coronaviridae* family genera was completed in Exp3. This experiment has a much smaller number of samples, the four genera: *Alphacoronavirus*, *Betacoronavirus*, *Deltacoronavirus*, and *Gammacoronavirus*, in which SARS-CoV-2 is a representative of the second. *Betacoronavirus* has 120 samples, and *Gammacoronavirus* has 10 samples, as in Figure 8. Then, as in the previous experiments, we take 10 random samples from each of these in Dataset2 and form Dataset5, which will be used for training and validation. The confusion matrix associated with Exp 3 is presented in Figure 9.

Due to the low number of samples, any classification error leads to a decrease in the classification accuracy, which leads us to believe that, with a more robust dataset, we would have a higher accuracy for the genus experiment. The biggest error was between *Gammacoronavirus* and *Betacoronavirus*.

**Figure 10:**
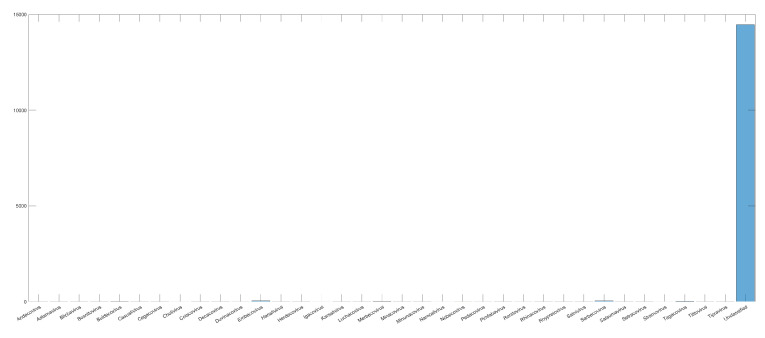
Histogram of sample distribution inside the *Betacoronavirus* subgenus. The number of samples without classification (Unclassified) is more than 15,000 times bigger than the ones with a defined subgenus.

We have a problem similar to that of Exp3 when performing Exp4, which is that we have few samples in each family. Another problem is that there are a lot of unclassified samples within the *(Betacoronavirus)* subgenus, as we can see in Figure 10, which leads us to few representative samples.

**Figure 11:**
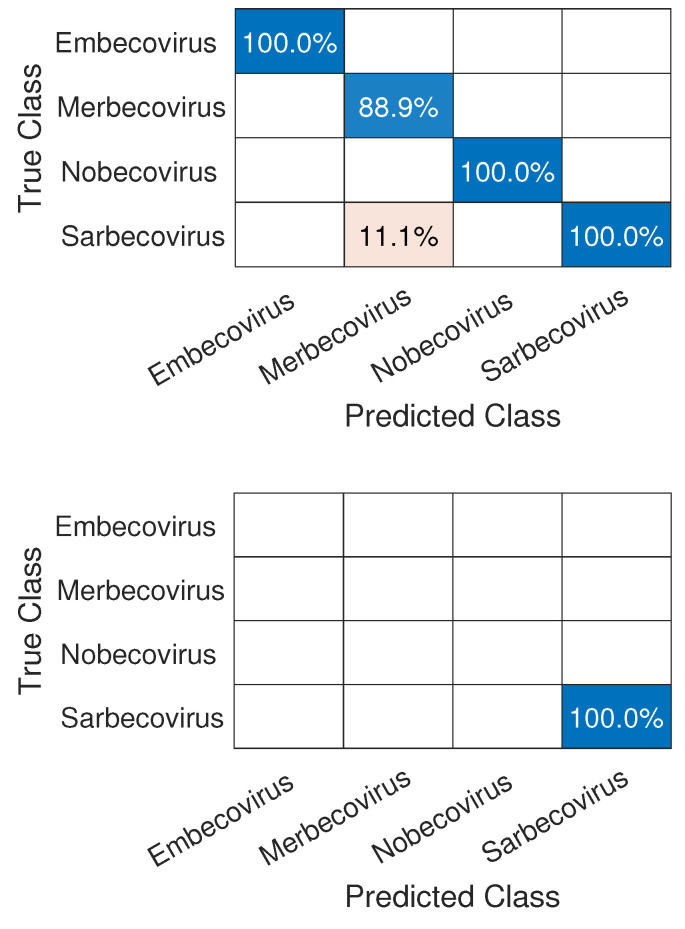
Confusion matrices for CNN validation using the subgenus samples within the *Betacoronavirus* genus (**top**) and, for SARS-CoV-2 classification, in its correct subgenus, *Sarbecovirus* (**bottom**).

As in the other experiments, we will also normalize the number of samples by the amount of the smallest subgenus, forming Dataset6 from Dataset 2, *Norbecovirus*, which leads to eight samples in each of these for training and validation. As a result, which we can see in Figure 11, we obtained 100% accuracy in the training and validation, except for *Merbecovirus* and, for the test carried out with the SARS-CoV-2 samples, we obtained an accuracy of 100% in its classification into its corresponding subgenus *(Sarbecovirus)*.

In Table 3, we have the values of the average training accuracy, the average training error, and their respective standard deviations for all the experiments performed, where we can observe that for the experiments between kingdoms, families, and genera, the results were quite similar, around 92%; in the case of subgenera, in which the similarity among all viruses is higher, the result increases to 97.5%.

### 3.2. Related Works Comparison

The most recent works to have used deep learning in viral classification are divided into two types of metrics used to verify their results. These works, presented in [41,42,47], use the model’s accuracy as the model proposed in this work and [39,40] use the area under the receiver operating characteristics (AUROC) curve.

Regarding the works associated with accuracy metric, in [47], a CNN was used to classify viruses, such as Influenza, Dengue, HIV, and Hepatitis, with an accuracy of at least 98%, reaching 100% for the Dengue virus. On the other hand, the work presented in [47] uses the complete sequence (without mapping) as the CNN input, and this constraint forces the creation of several different models for different viral families and considerably increases the computational effort in the first layers. Using the tree-shaped model, with a CNN responsible for classifying the viral RNA, [42], they tried to classify the genome into orders, families, and genera. In addition, the RNA could be classified as non-viral. For the genera and families, they obtained 86% and 79% accuracy. In the scheme proposed in this work, the classification accuracies in families and genera were more significant than 92%, demonstrating a better capacity for viral classification. Using the complete viral genome and a convolutional three-layer CNN [41] achieved over 96% accuracy, which is an excellent result, but their dataset contains around 400 samples, of which only 50 are SARS-CoV-2, a deficient number, which can lead to generalization problems across the model.

Regarding the works that use AUROC as a metric, [39] uses a CNN that receives a metagenomic dataset as the input and achieved an AUROC of 0.923, but this value decreased to approximately 0.75 when randomly generated virus sequences that were not used in training were used as test set; this can be a sign of a generalization problem. Finally, the additional utilization of metagenomics data, especially from prokaryotes, [40] reached an AUROC of 0.966, which leads to a result superior to that of [39]. However, taking into account that the maximum size of the sequences used in this work was 3000 bp, this may be a problem because a virus such as SARS-CoV-2, one of the most targeted at the moment for studies, has approximately 30 kbp.

The CNN proposed in this work achieved accuracies similar to or greater than those of the works mentioned above. In addition to having a good result in classifying SARS-CoV-2 into its respective taxon and using the complete viral genome as the input in an image format, the genome size does not affect the computational cost of training. Table 4 summarizes the related comparison between more recent works involving deep learning and viral classification.

## 4. Conclusions

The convolutional neural network proved to be an effective way to correctly classify viruses, especially SARS-CoV-2, into their realms, families, genera, and subgenera. Despite the promising results (above a 92% accuracy for the general classification and 100% for the new coronavirus) that prove this model to have a similar or greater accuracy to other works and a good generalization capacity, we believe that new modes of an image representation of the viral genetic code may help increase these values; this is the future path of the proposed work.

## Figures and Tables

**Table 1:** Contents of datasets used in this work.

Datasets	Content	Number of Classes
Dataset1	1553 samples	SARS-CoV-2 species
Dataset2	14,684 samples	4 realms 95 families 1160 genera 36 subgenera

**Table 2:** CNN architectures tested in classifying the 14,684 viruses in their realms. The architecture chosen in this experiment was used in the other experiments in this paper.

Mini-Batch Size	1st Convolutional Filter	2nd Convolutional Filter	3rd Convolutional Filter	Stride Size	*K*-Fold Size	CNN Accuracy (%)	Standard Deviation
128	7×7	5×5	3×3	1	10	92.5	2.149
128	7×7	5×5	3×3	2	10	92.37	2.1205
64	7×7	5×5	3×3	2	10	92.1875	1.9151
128	5×5	5×5	3×3	2	10	91.9375	2.3096
128	5×5	5×5	3×3	2	5	91.8125	1.4218

**Table 3:** Validation accuracy and validation error results for the 4 experiments performed.

Experiment	Validation Accuracy (Mean ± Standard Deviation)	Validation Error (Mean ± Standard Deviation)
Exp1	91.94±2.62	0.40±0.20
Exp2	92.00±2.26	0.31±0.14
Exp3	92.70±2.78	0.21±0.09
Exp4	92.50±7.90	0.09±0.19

**Table 4:** Comparison between more recent works involving deep learning and viral classification.

Reference	Maximum Sequence Size (bp)	Dataset Size (Numbers of Sequences)	Number of Convolutional Layers	Accuracy	AUROC
Fabijańska and	24,751	665,353	5	97.7–100%	–
Grabowski (2019) [47]					
Ren et al. (2020) [40]	3000	1,735,440	1	–	0.966
Tampuue et al. (2019) [39]	300	–	2	–	0.923
Lopez-Rincon et al. (2020) [41]	31,029	553	3	96%	–
Shang and Sun (2020) [42]	250	438,072	4	79–96%	-
This work	2,473,870	14,684	3	91.94%	–

## Data Availability

All k-mers image representation data of the virus created or used during this study are available from the k-mers 1D, and 2D representation dataset of SARS-CoV-2 nucleotide sequences https://doi.org/10.17632/F5Y9CGGNXY.2 [44].
